# OL-EDA-ID Syndrome: a Novel Hypomorphic NEMO Mutation Associated with a Severe Clinical Presentation and Transient HLH

**DOI:** 10.1007/s10875-016-0350-x

**Published:** 2016-11-12

**Authors:** Silvia Ricci, Francesca Romano, Francesco Nieddu, Capucine Picard, Chiara Azzari

**Affiliations:** 1 Department of Pediatric Immunology, Jeffrey Modell Center for Primary Immunodeficiencies, University of Florence and Anna Meyer Children’s University Hospital, viale Pieraccini 24, 50139 Florence, Italy; 2 St Giles Laboratory of Human Genetics Infectious Diseases, Rockefeller Branch, The Rockefeller University, New York, NY 10065 USA; 3 Laboratory of Human Genetics Infectious Diseases, Necker Branch, INSERM U1163, Imagine Institute, Paris Descartes University, Paris, France; 4 Pediatric Hematology-Immunology Unit, AP-HO, Necker Hospital for Sick Children, Paris, France; 5 Center for the Study of Primary Immunodeficiences, AP-HP, Necker Hospital for Sick Children, Paris, France

To the Editor:

## Background

NEMO deficiency syndrome is a rare and heterogeneous condition that presents in early infancy. The phenotypic spectrum is broad ranging from hypogammaglobulinemia and mild ectodermal signs to OL-EDA-ID syndrome, the most severe phenotype for IKBKG hypomorphic mutations [1].

NF-κB is a transcriptional factor involved in many signaling pathways, and NEMO plays a key role in its activation. Many human hypomorphic mutations in IKBKG have been described involving IL-1 family protein receptors, TLR, VEGFR-3, RANK, the ectodysplasin-A receptor, CD40, and TNF receptor signals. It is known that the regulation of genes essential for cell adhesion, cell survival, immunoglobulin class switching, osteoclast function, and T and B cell development can be impaired but the knowledge of mechanisms that explain the relationship between genotype and phenotype is still incomplete.

## Case Report

**Fig. 1:**
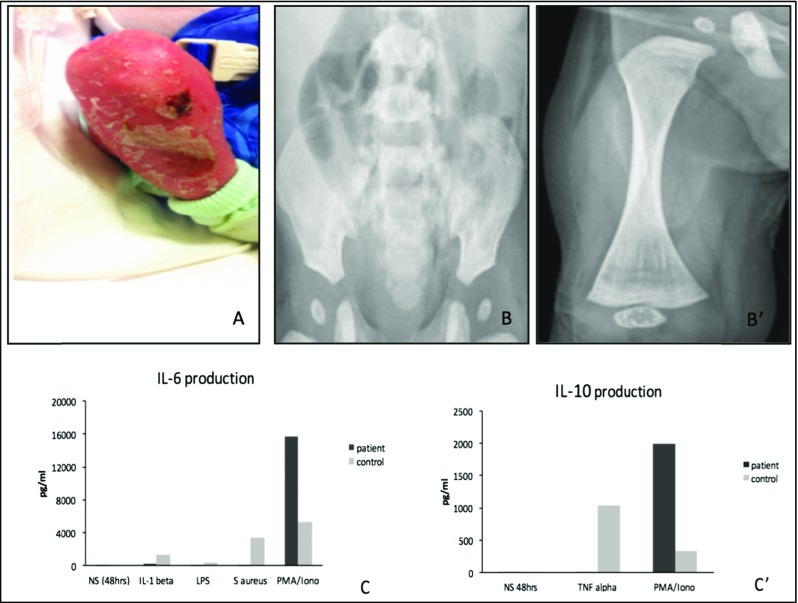
Right lower extremity with signs of ectodermal dysplasia (*A*). Radiological findings of osteopetrosis with increased bone intensity, bone-within-bone appearance of the iliac and ischial bones (*B*), and of femoral epiphyses (*B′*). Functional test evaluation performed by ELISA: impaired IL-6 production in patient after 48 h of activation of whole blood with IL-1β, LPS, and S aureus Cowan I (SAC) (*C*) and impaired IL-10 production after 48 h of activation with TNF-α. Increased IL-6 and IL-10 production after PMA/Ionomycin stimulation (*C′*). The results (*C* and *C′*) are representative of two tests

**Fig. 2:**
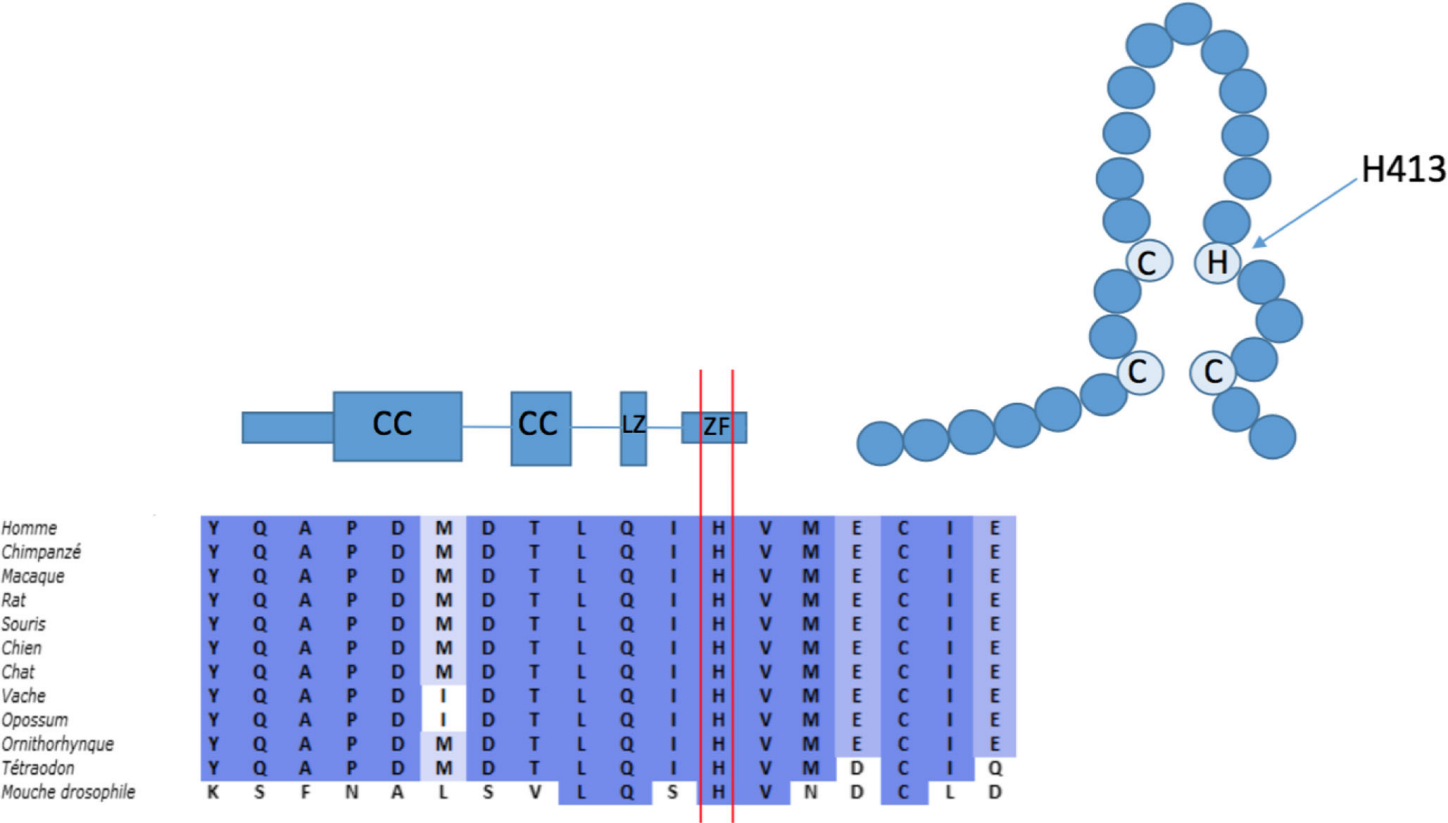
The NEMO gene structure and its zinc finger (*ZF*) which extends from amino acid residues 397 to 419. The three cysteine residues and the single histidine (*H413*) residue that coordinate a zinc ion are indicated. H413 is high conserved among different species (modified by Shifera AS *The zinc finger domain of IKKγ (NEMO) protein in health and disease* J. Cell. Mol. Med.)

We examined a 3-month-old infant with persistent diarrhea and failure to thrive. The male patient was born at term to healthy non-consanguineous Italian parents after an uncomplicated pregnancy. His umbilical separation was delayed. At admission, he had a temperature of 38 °C, CRP concentration of 41 mg/L, white blood cell count of 34.6 × 10^3^/μl, polymorphonuclear cell count of 14.11 × 10^3^/μl, lymphocytic cell count of 15.43 × 10^3^/μl, and hypereosinophilia (3.8 × 10^3^/μl). Adenovirus was detected in his stool sample. Physical examination revealed mild dysmorphic features with frontal bossing, micrognathia, hypoplastic nasal wings, and flattened alveolar margins. He presented signs of anhidrotic ectodermal dysplasia with fine and sparse hair, absent eyebrows, thin translucent skin with dry eczema, and hyperkeratosis (Fig. 1(A)). The absence of sweat glands at skin biopsy confirmed this hypothesis. Radiologic evaluation was performed and the images showed “bone-in-bone appearance” of the femoral, iliac, and ischial bones bilaterally, consistent with a diagnosis of osteopetrosis (Fig. 1(B, B′)). His general condition rapidly deteriorated with significant dyspnea, oliguria, and lethargy. He presented acute seizures. A head CT scan was negative. MRI was not performed. He was immediately admitted to the Pediatric Intensive Care Unit. On admission, he had leukocytosis (52.24 × 10^3^/μl) and the signs of secondary hemophagocytic lymphohistiocytosis with prolonged fever, hepatosplenomegaly, anemia (Hb 6.9 g/dl), elevated ferritinemia (37,300 ng/ml), LDH (2105 U/L), low fibrinogen (93 mg/dl), and hypertriglyceridemia (700 mg/dl). Conventional respiratory and circulatory support with inotropes was necessary. A thoracic scan image was concordant with interstitial pneumonitis. *Pneumocystis jirovecii* and CMV DNA were identified in the bronchoalveolar lavage by PCR. The patient was successfully treated with meropenem, trimethoprim/sulfamethoxazole, and ganciclovir. The signs of concomitant macrophage activation syndrome were gradually normalized under systemic corticosteroid therapy. During his prolonged hospitalization, the patient also displayed a transient and inconstant lymphedema of the lower limbs. Immunological assay was performed at 4 months of life. The patient presented severe hypogammaglobulinemia with IgG (1.73 g/L with normal range 2.22–8.46 g/L), IgA (0.01 g/L with normal range 0.06–0.6 g/L), and IgM (0.04 g/L with normal range 0.28–0.39 g/L). TRECs and KRECs were normal. Flow cytometry immunophenotyping revealed low NK and B memory cell counts (Table 1). NK cell functional activity (CD107a expression) could not be determined due to the low number of NK cells. Proliferative T cell responses to mitogenic stimuli were normal (Table 1). We performed an analysis of the IKBKG sequence (NM_001099856.3) from both genomic DNA and cDNA and we identified a novel missense mutation c.1238A>G (p.H413R) within exon 10 on the zinc finger domain. The substituted histidine is highly conserved among different species (Fig. 2). Using a bioinformatic system (Polyphen and SIFT-Sort Intolerant From Tolerant), the new mutation is predicted to be damaging with a score of 0.99 (sensitivity 0.09; specificity 0.99) and to affect protein function with a score of 0.00 (intolerant), respectively. The CADD score was also elevated (22.9). We performed a careful dermatological examination of the patient’s mother, we did not observe any of the nail, hair, dental, or skin findings typical of *incontinentia pigmenti*. Moreover, there was no history of dermatological problems in the family. Maternal sequencing on genomic and cDNA revealed the WT IKBKG on both alleles. We evaluated the patient’s blood cells after activation with TNF-α, IL-1β, and other agonists of TLRs. The response of the patient’s blood cells to IL-1β, LPS (agonist of TLR-4), and SAC was abnormal, in terms of IL-6 production (Fig. 1(C)). Moreover, the response to TNF-α was impaired in terms of IL-10 production (Fig. 1(C′)). IL-6 and IL-10 production was measured by enzyme-linked immunosorbent assay (ELISA) after 48 h of activation. For persistent bloody diarrhea, hypoproteinemia and feeding intolerance parenteral nutrition was necessary. Colon biopsy showed macroscopic signs of enterocolitis with diffuse eosinophilic infiltrates in the lamina propria of the colon. Despite IV antimicrobial prophylaxis and regular infusions of IV immunoglobulin, at 10 months of age, the patient was readmitted presenting a new episode of catheter-associated bacteremia from *E. coli*. At 13 months of age, the patient underwent a myeloablative conditioning regimen consisting of thiotepa, treosulfan, and fludarabine followed by haploidentical stem cell transplantation with TCR-alpha/beta and CD19 depletion. He received anti-thymocyte globulin and mycophenolate mofetil with a complete antimicrobial prophylaxis as a prevention of acute GVHD and infectious complications. He died unexpectedly from acute respiratory distress and sepsis due to multiresistant *Pseudomonas aeruginosa* 6 days post HSCT.

**Table 1:** Patient’s lymphocyte phenotyping and T-lymphocytes proliferation in response to mitogens, performed by FACS at 5 months of life

Cell population	%	Cells/μl
Lymphocytes		7945
T-Lymphocytes	75	7282
CD3+CD4+	66	6369
CD3+CD8+	9	881
CD45+CD3+CD4+CD31+	24	1528
CD45+CD3+CD4+CD45RO+	54	3439
CD45+CD3+CD4+CD45RA+	46	2930
CD45+CD3+CD4+CD45RA+CD31+	39	2485
B-Lymphocytes	5	481
CD27+	2	10
CD27+IgM+IgD+	76	9
CD27+IgM−IgD−	24	2
CD3−CD16+CD56+	2	206
CD3−CD19+CD40+	99	1906
CD3+CD8−CD40L	97	6175
T-lymphocytes proliferation assay		
CFSE+ w/o stimulus	98%	
PHA (10 μM)	77%	
Anti-CD3 antibody and IL-2 (30 U/ml)	85%

## Discussion and Conclusion

We report a case characterized by a severe clinical presentation and fatal outcome associated with a genotype never previously described. The clinical features of the case, in particular the EDA signs and the life-threatening infection, led us to consider a diagnosis of a severe form of NEMO deficiency syndrome. Indeed, the association of osteopetrosis/lymphedema and EDA-ID is indicative of the most severe phenotype. The immunological assay was completely compatible with previously reported cases. As previously described, the CD40-CD40L signaling on the dendritic cells and B cells involves NEMO in NF-κB activation. Impaired CD40 signaling in pulmonary dendritic cells may result in a major susceptibility to fungi infections similar to patients with hyper IgM syndrome [2]. Moreover, the patient displayed a very low NK cell count which can explain his increased susceptibility to the herpes virus group, including CMV [3]. The use of HSCT in NEMO deficiency syndrome remains controversial and few data are available concerning the transplantation of a patient with a novel NEMO mutation [5]. Despite the known intrinsic difficulties with engraftment for these patients, having evaluated the severity of his life-threatening infections and their sequelae, we decided that HSCT was the only therapeutic option. A similar case of OL-EDA-ID syndrome caused by the substitution of a stop codon with a tryptophan (X420W) on the zinc finger region in the NEMO gene has been reported. This substitution changes the length of the final protein, resulting 27 amino acids longer than the WT protein [2]. Here, we describe a completely different genotype, a novel missense mutation, which substitutes histidine for arginine at amino acid 413 on the zinc finger domain, and which resulted in an equally severe phenotype. In addition, this case is characterized by the precocious and serious development of intestinal failure and hemophagocytic lymphohistiocytosis. These clinical signs have previously been described but never together in association with a severe OL-EDA-ID phenotype. Recently, a variation involving the same amino acid—p.H413Y—causing IP syndrome in a female patient with a random X-inactivation has been reported [4]. In 2008, Cordier et al. presented a complete analysis of the structural and functional properties of the ZF motif of NEMO. The integrity of the tetrahedral zinc coordination site formed by H413 and another three cysteine residues determines the ββα scaffold of NEMO ZF (Fig. 2). In our case, the highly conserved H413 is substituted by an arginine; this implies an impaired stability of the ZF fold which may alter its protein recognition abilities. It appears that the disruption of C-terminus of NEMO gene, caused by nonsense mutation or by a missense mutation of a key conjugating residue, lead to similar severe phenotype. Moreover, functional complementation assays using the patient’s mononuclear cells showed that the H413R NEMO mutation leads to a strong defect of LPS, IL-1β, and TNF-α-induced NF-κB activation, as compared to WT NEMO (Fig. 1(C, C′)). Interestingly, cytokine production is higher in patient than in control after PMA/ionomycin stimulation. Zilberman-Rudenko et al. have recently described a distinct group of patient with inflammatory symptoms caused by gain-of-function C-terminus deletion which confers increased responsiveness to innate immune stimuli. Regulated activation of NF-kB transcription factors family is important in immune cell function and inflammatory responses. Our case represents an example of immunodysregulation characterized for clinical and molecular features of immunodeficiency and auto-inflammation. We are aware that more studies are necessary to establish all pathogenetic mechanisms of this point mutation; unfortunately, we did not obtain the family’s consent for a second skin biopsy or for other investigations. In conclusion, we report a novel missense mutation responsible for one of the most complex and severe clinical presentations of reported NEMO deficiency cases. It appears that the disruption of C-terminus of NEMO gene, caused by nonsense mutation or by a missense mutation of a key conjugating residue, lead to similar severe phenotype. We recommend that each novel variation is described and submitted to public databases because any additional data will provide insight into genotype-phenotype correlation and will improve patient care for infants with NEMO deficiency syndrome.

## Abbreviations

NF-kB: Nuclear factor kappa-light-chain-enhancer of activated B cells
NEMO: NF-kappa B Essential Modulator
IKBKG: Inhibitor of kappa light polypeptide gene enhancer in B cells, kinase gamma
OL-EDA-ID: Osteopetrosis and lymphedema-anhidrotic ectodermal dysplasia with immunodeficiency
HLH: Hemophagocytic Lymphohistiocytosis
IL: Interleukin
TLR: Toll like receptor
VEGFR-3: Vascular endothelial growth factor receptor-3
RANK: Receptor activator of NF-κB
CRP: C-Reactive protein
PCR: Polymerase chain reaction
HSCT: Hematopoietic stem cell transplantation
CADD: Combined annotation dependent depletion
TREC: T cell receptor excision circle
LPS: Lipopolysaccharide
SAC: Staphylococcus aureus Cowan I
TNF-α: Tumor necrosis factor-α
PMA: Phorbol myristate acetate
GVHD: Graft versus host disease
